# Evaluation of the Slovenian Breast Cancer Screening Programme: Years of Life Gained and Avoided Deaths

**DOI:** 10.3390/cancers17050742

**Published:** 2025-02-22

**Authors:** Sonja Tomšič, Vesna Zadnik, Maja Pohar Perme, Tina Žagar, Katja Jarm, Bor Vratanar

**Affiliations:** 1 Epidemiology and Cancer Registry, Institute of Oncology Ljubljana, 1000 Ljubljana, Slovenia; vzadnik@onko-i.si (V.Z.); tzagar@onko-i.si (T.Ž.); 2 Institute for Biostatistics and Medical Informatics, Faculty of Medicine, University of Ljubljana, 1000 Ljubljana, Slovenia; maja.pohar@mf.uni-lj.si; 3 Slovenian Breast Cancer Screening Programme DORA, Institute of Oncology Ljubljana, 1000 Ljubljana, Slovenia; kjarm@onko-i.si

**Keywords:** breast cancer, organized screening, survival analysis, years of life gained, avoided deaths

## Abstract

**Background**: Most commonly, mortality is used for evaluation of organized breast cancer screening programmes. Survival analyses are also being employed to provide more timely assessment. A novel approach has been introduced for calculating survival using Slovenian data. **Methods**: Breast cancer cases from the Slovenian Cancer Registry between 2008–2021 in women aged 50 to 72 were included, supplemented by data on the screening history from the screening registry. The comparison was made in two scenarios—invited or not invited to the screening. Survival, years of life gained, and number of avoided deaths over 10 years were calculated and standardized. The comparison was adjusted for confounding covariates, and a biological tumour growth model was used to adjust the survival of the invited group for lead time bias and over-detection. **Results**: A total of 9392 breast cancer cases were included in the study. Estimated median lead time for screen-detected cases was 1.23 years. The survival probability accounting for lead time bias and covariates of cancers patients invited to the programme was 4.3 percentage points higher than the not invited (0.81 vs. 0.76). Standardized to 100,000 women in the 2-year period, this would result in 22 avoided deaths. On average, the invited cancer patient lived 0.22 years longer, which amounts to a total of 114 years of life gained. **Conclusions**: The results show the overall benefit of the breast cancer screening programme. The standardized results enable cross-country comparisons. The calculation of years of life gained and avoided deaths can provide additional opportunities for communicating the results to non-expert populations.

## Simple Summary

Public health interventions should evaluate their effect on the population. Measuring outcomes in organized cancer screenings is not straightforward, since expected reduction in mortality has drawbacks such as long observation time, quality of data, and the relationship between the intervention and the outcome. Researchers are exploring the use of survival, which is available sooner, but has limitations such as lead time bias, length time bias, and over-detection. Slovenian researchers have used a novel approach addressing the known biases comparing the survival between invited and not-invited women and providing estimations of years of life gained and deaths avoided for an easier interpretation for policy-makers. The results show that after 10 years the invited women have a 4.3 percentage points higher survival probability than the not invited, resulting in 22 avoided deaths per 100,000, amounting to a total of 114 years of life gained. The standardized results allow cross-country comparisons.

## 1. Introduction

Breast cancer, which is the most common cancer in women worldwide, is one of the cancers where there is enough evidence of organized screening being beneficial for women attending it, and its introduction is thus recommended [1,2]. The primary aim of screening is early detection and treatment to reduce breast cancer mortality and improve quality of life [3,4].

All screening programmes need to monitor their quality and effectiveness to ensure that healthcare resources are allocated efficiently. Most commonly, mortality is used as the primary endpoint outcome. Its major drawback is that especially in cancers with good survival, which breast cancer is, the evaluation needs decades [5]. Additionally, there is uncertainty regarding how much of the observed improvement can be attributed to screening, improvements in treatment [6,7,8,9], or other health care interventions affecting mortality due to breast cancer, along with concerns about the quality of data on causes of death [10,11]. Therefore, in the effort to obtain more timely and relevant assessment of screening effectiveness, epidemiologists can instead turn to survival analysis. However, depending on the design of the study and the choice of comparison to be studied, there are at least three biases that have been described in relation to measurement of survival as the main outcome. Lead time bias refers to the phenomenon whereby the timing of diagnosis is shifted to an earlier date, resulting in an apparent increase in survival time measured from the point of diagnosis for cases detected by screening, even if total length of life remains unchanged [12]. Length bias arises when comparing patients diagnosed based on screening with any relevant control group. It occurs because screen-detected cases do not represent a random subsample of all cases; cases with a longer pre-clinical phase are more likely to be detected than those with faster-growing tumours [12]. Finally, there is a bias due to over-detection where due to the long natural trajectory of the disease some of the lesions detected by the screening programme would not cause any cancer symptoms in the course of the individual’s lifetime or would not be observed during the study period. Thus, the true benefit of identifying such pre-clinical lesions through screening may be much smaller than is perceived [12]. Many approaches have already been suggested to correct for different biases [13,14,15,16,17,18,19,20,21]. Further possibilities of how to minimize the biases when comparing survival probabilities related to organized screening are being explored. Recently, Vratanar and Pohar Perme introduced a novel approach that helps in addressing all of the aforementioned biases and hence provides a more accurate and fair estimation of survival [22].

In Slovenia, the national organized breast cancer screening programme was launched in 2008, inviting women between 50 and 69 years old to digital mammography screening every two years, with all procedures established according to European guidelines for quality assurance in breast cancer screening and diagnosis. Initially, the organized breast cancer screening programme was limited to women in central Slovenia; by 2018, it was gradually extended to cover all municipalities [23,24]. This phased implementation constitutes a natural experiment, with invited women serving as the experimental group and uninvited women as the control group. In our previous study, we explored whether the implementation of the national screening programme is reflected in better survival of breast cancer cases diagnosed at screening. Stratification by stage was applied in that study to reduce the lead time bias and the survival probability was significantly higher in the invited-to-screening group [25]. Still, the control group (not invited to screening) differed in many aspects to the screened group and further analysis which would allow us to make more robust causal statements is warranted.

This study aims to improve the previous evaluation of the Slovenian breast cancer screening programme in two aspects: first, by applying improved statistical methods to analyse the data more accurately; and second, by introducing new metrics that are more understandable and easier to interpret for the general public. In the analysis, we compare all cancer patients invited to the programme with those not invited, and employ a biological tumour growth model [21] to reduce lead time bias. Our approach ensures that the comparison is fair and bias-free. The endpoint outcomes include years of life gained and the number of avoidable deaths; both metrics are standardized to enable cross-country comparisons.

## 2. Materials and Methods

### 2.1. Dataset

For this epidemiological study with predictive modelling, the data on cancer cases were acquired from the population-based Slovenian Cancer Registry. The initial dataset consists of 9416 invasive breast cancers recorded in the cancer registry from 1 January 2008 to 1 January 2021 (code C50 according to ICD-10 classification; carcinoma in situ was not included in the analysis) diagnosed in women aged between 50 and 72. Of these, 21 were classified as death-certificate-only cases, which were excluded. Additionally, three screen-detected cases with missing tumour diameter values were also removed. This resulted in 9392 cancer cases that were included in the subsequent analysis. We included cases occurring in women with the upper age limit of 72 years, which is two years after the last inclusion in the screening programme; this is to observe the effect that screening has on the eligible population beyond the screening age due to the lead time and to include also the interval cases that would occur after the last screening round at the age 69 years. The acquired data for each case include the following variables: date of birth, date of cancer diagnosis, date of death, or date of last follow-up (last update on 1 October 2023), status at the end of the follow-up (alive, dead, lost to follow-up), cause of death, and socio-economic index. The socio-economic index was determined using the Slovenian version of the European Deprivation Index (SI-EDI) for the year 2011. The index was measured at the level of the National Assembly Polling Stations, which represents the smallest administrative geographic units with available population data [26]. The SI-EDI was categorized into quintiles, where each quintile represents a segment that includes 20% of the data, ordered from the most affluent (group 1) to the most deprived (group 5). Each cancer patient was assigned to a group based on their address at the time of diagnosis.

In addition, we collected data from the Slovenian national breast cancer screening registry [23]. These include invitation status, screening history, and tumour size at cancer diagnosis. Detection mode was determined from the data and categorized into three groups: screen-detected cases, interval cases, and cancers in non-attenders (never screened or diagnosed based on symptoms more than 2.5 years from the last screening). The definition of interval cancer has been adjusted to include a period up to 2.5 years, when more than 98% of eligible women should be re-invited to screening (instead of 2 years, which corresponds to the proposed length of a screening interval) [27] to accommodate possible slight delays in screening appointments (most often caused by women’s request). Interval cancer patients were determined solely on the time interval from the last performed mammography, even in women who did not respond to the next invitation to screening mammography, which could be in the interval 20 to 30 months after previous mammography.

We also obtained background population data (counts) from the Statistical Office of the Republic of Slovenia for women aged 50 to 72, spanning each year from 2008 to 2021, for standardization purposes.

### 2.2. Statistical Analysis

#### 2.2.1. Experimental and Projection Periods

The estimated effectiveness of cancer screening programmes in their initial stages is context-specific, influenced by fluctuating participation rates and a large pool of potentially screen-detectable cancers, which leads to an unstable ratio of screen-detected to interval cases compared to later periods. For our results to be more generalizable, we divided the study into two distinct periods: the Experimental period and the Projection period.

**Figure 1:**
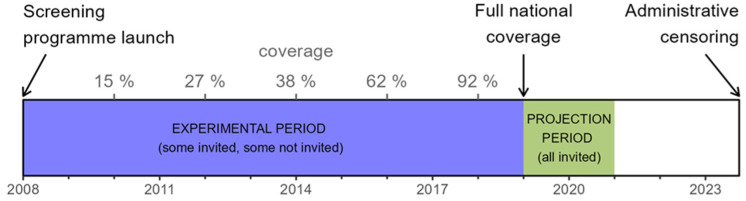
The Experimental and Projection periods, along with the coverage rate of the Slovenian breast cancer screening programme.

The Experimental period aligns with the initial phase of the programme (from 2008 to 2019), during which the programme gradually expanded to cover the entire country (the expansion rate is shown in Figure 1). During this period, we collected data on both cancer patients invited to the programme and those not invited, allowing for comparative analyses of survival probabilities between the two groups. Since our data come from a natural experiment, governed by the politics of breast cancer screening introduction, the two groups are not directly comparable. The programme initially prioritized older women and more urbanized regions (with higher socio-economic statuses), affecting the distribution of demographic variables.

The Projection period takes place after the programme has expanded to the whole country (from 2019 to 2021; see also Figure 1). During this period, we have data for cancer patients invited to the programme, but not for non-invited cases, as the programme achieved nationwide coverage. This period is expected to be more representative of future screening effectiveness, as the programme had been well-established by this time.

#### 2.2.2. Addressing the Lead Time Bias, Length Bias, and Over-Detection Biases

To adjust for lead time bias in our analysis, we estimated lead time values for each individual based on the biological tumour growth model [21]. We used model parameters estimated based on the Swedish dataset of a breast cancer screening programme as given in [21]. Following the approach proposed by Abrahamsson et al. [21], we sampled lead time for each Slovenian woman with cancer detected on a screening test based on her tumour size and screening history, leading to an individual lead time estimate that was used in further calculations. For interval cases and non-attenders, lead time was set to zero. Person-specific lead times were subsequently used to predict the age at hypothetical symptomatic detection.

**Figure 2:**
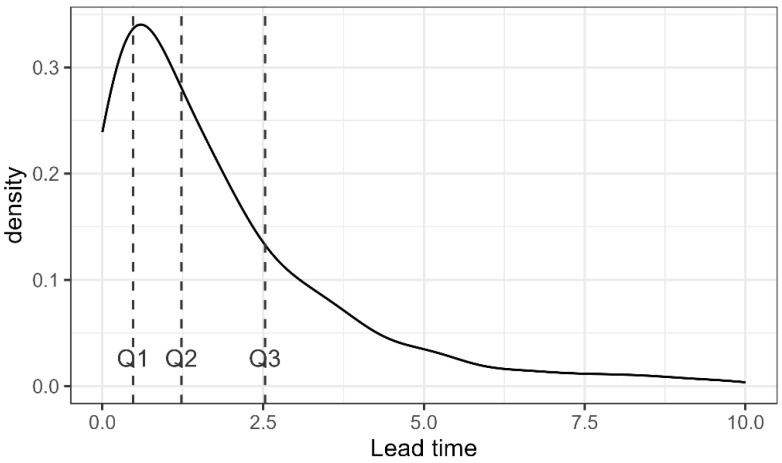
Distribution of lead time calculated on the data from the Experimental period (one run). The vertical lines represent 1st, 2nd, and 3rd quartile.

The median lead time for screen-detected cases was estimated to be 1.23 years (1st quartile = 0.48 years, 3rd quartile = 2.53 years). Figure 2 presents the estimated distribution of lead time, calculated based on our covariates and Swedish parameters. The survival time was computed by subtracting the age at predicted symptomatic detection from the age at death.

Women who died due to other causes prior to the predicted age of symptomatic detection were flagged as over-detected cases. The same was done if the predicted age of symptomatic detection was beyond 72 years as the study population was limited to cancer patients aged between 50 and 72 years. In our analysis, there were 10 cases of breast cancer that were over-detected per 100,000 women adjusted for covariates.

To avoid length time bias, we compared all cancer cases invited to the programme against all cancer cases not invited to the programme since similar case-mix of tumours is present in both populations.

#### 2.2.3. Confounders and Scenarios

Because our data come from a natural experiment, the two groups—invited and not invited—observed during the Experimental period are not directly comparable (see Section 2.2.1). To address this issue, we used regression adjustment via a Cox proportional hazards model to align the covariate distributions of both groups to match the distribution observed in the Projection period. This approach estimates survival probabilities as if both groups had the same covariate distribution as in the Projection period. This alignment serves two purposes: first, it balances the two groups, making them directly comparable; and second, it enables predictions for the Projection period. A single Cox proportional hazards model was used to adjust the survival of each group with respect to confounding covariates. In this model, we included the following covariates: SI-EDI (groups from 1 to 5), detection mode (a new category “non-invited cancer” was created to describe cancer patients not invited to the programme), and lead-time-adjusted age and date of diagnosis. Both SI-EDI and detection mode were entered into the model as indicator variables (the reference category was 1 for SI-EDI and “non-invited cancer” for detection mode). The relationship between hazard and age, as well as the date of cancer diagnosis, was modelled using restricted cubic splines.

The parameters of the model were estimated based on the data (lead-time-adjusted) from both phases to reduce the variance of our estimates and to minimize extrapolation. This model was subsequently used to predict the survival of each (sub)group during the Projection period under two scenarios: Scenario A examines the situation where all cancer patients are invited to the programme, and Scenario B considers the situation where none are invited. The survival of each subgroup in Scenario A (screen-detected, interval, and non-attenders) was predicted using the distribution of covariates, adjusted for lead time, specific to each subgroup from the Projection period. The survival for all cancer patients in Scenario A was estimated by weighting the survival of each subgroup based on their proportions observed during the Projection period (and adjusted for over-detection) and summing these values at each time point. For cancer patients in Scenario B, the survival was predicted based on the covariate distribution (adjusted for lead time) of invited cases during the Projection period, with detection mode indicators set to zero.

#### 2.2.4. Calculations of Years Gained and Deaths Avoided in the Two Scenarios

Our final goal was to estimate the number of years gained and deaths avoided per 100,000 women invited to the programme (Scenario A) compared to a counterfactual scenario where they were not invited (Scenario B). The population at risk, as observed during the Projection period, was defined to represent our target population. The main results are presented in the form of flowchart and further visually represented in an infographic. Below, the calculations for each labelled step in the flowchart are explained in detail:

- The attendance rate in Scenario A was estimated by dividing the total number of screenings by the total number of invitations in the Projection period;
- The number of cancer patients in each subgroup (screen-detected cases, interval cases, and non-attenders) in Scenario A was estimated based on the data from the Projection period and normalizing the counts to a population size of 100,000. All other individuals who did not develop cancer are excluded from the flowchart to improve visibility;
- The number of cancer patients in Scenario B was calculated by subtracting the number of over-detected cases from the total number of cases in Scenario A. The number of over-detected cases in the Projection period was estimated as described in Section 2.2.2. All other individuals who did not develop cancer are excluded from the flowchart to improve visibility;
- Observed 10-years survival was estimated using the Kaplan–Meier estimator, and the corresponding 10-years restricted mean survival time (RMST) was estimated as the area under the survival curve from 0 to 10 years (measured in years).
- The survival of screen-detected cases was adjusted for lead time (and over-detection) as described in Section 2.2.2;
- The 10-year survival and RMST during the Projection period were adjusted for covariates (and lead time), and predicted;
- The estimated 10-year survival probabilities and corresponding RMSTs for each subgroup (screen-detected cases, interval cases, and non-attenders) were weighted and aggregated. The weights, estimated as detailed in step (b), were adjusted for over-detection;
- The survival benefit was estimated by subtracting the estimated 10-year survival in Scenario B from that in Scenario A. To estimate the number of avoidable deaths, we first estimated the number of deaths in each scenario by multiplying the proportion of individuals who died (1—survival) within 10 years of cancer diagnosis (lead time adjusted) by the total number of patients in Scenario B. The number of avoidable deaths was then calculated as the difference between the number of deaths in Scenario B and Scenario A;
- The years of life gained (YLG) per cancer patient were calculated by subtracting the RMST of Scenario B from that of Scenario A. The total YLG was determined by multiplying the YLG per case by the number of cancer patients in Scenario B.

#### 2.2.5. Addressing Statistical Uncertainty and Software Tools Used

Our analysis contains two levels of statistical uncertainty: the first arises from sampling, and the second from the stochastic generation of lead time values. To address the first source, 95% confidence intervals were calculated using bootstrap method. We generated 10,000 bootstrap samples, and the limits of the 95% confidence intervals were determined by approximating the resulting sampling distribution with a normal distribution. To address the second source, lead time was sampled 100 times for each individual within each bootstrapped sample and the results were averaged.

All statistical analyses were performed using the R programming language version 4.2.2 [28].

## 3. Results

### 3.1. Comparison of Study Populations in Two Periods

**Figure 3:**
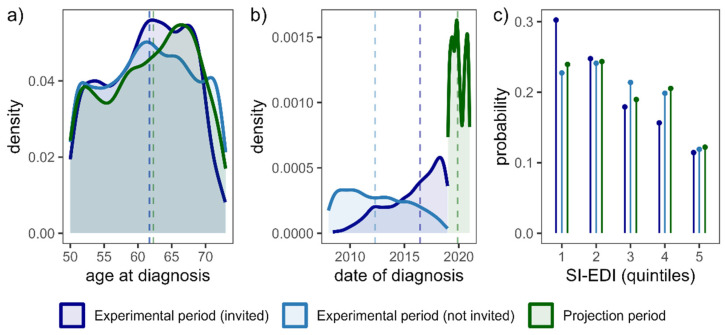
The distribution of confounders for cases invited to the programme and cases not invited to the programme during the Experimental period and for cases invited to the programme during the Projection period. The dashed vertical lines denote median values for each group. (**a**)—distribution of age at diagnosis, (**b**)—distribution of date of diagnosis, and (**c**)—distribution of socio-economic conditions represented by SI-EDI (Slovenian European Deprivation Index), which is segmented into quintiles, ordered from first quintile (most affluent) to fifth quintile (least affluent).

In total, there were 7733 cancer patients (1842 deaths) during the Experimental period, with 2702 (403 deaths) invited to the programme and 5031 (1439 deaths) not invited. During the Projection period, there were 1659 cancer patients (118 deaths) invited to the programme. In Figure 3, we compare the distribution of relevant confounding variables between cancer patients invited to the programme and those not invited during the Experimental period. As explained previously, the gradual and uneven implementation of the programme leads to significant differences in the distribution of these variables between the two groups. These discrepancies were adjusted for in the subsequent analysis to align with the distribution of cancer patients invited to the programme during the Projection period (indicated by the green line in Figure 3).

### 3.2. Survival Analysis

**Figure 4:**
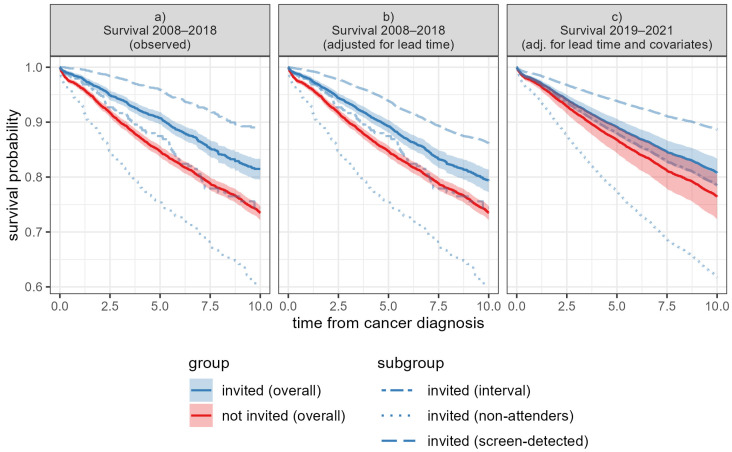
Comparison of survival of breast cancer patients between invited and not-invited groups in the Experimental and Projected periods, adjusted for lead time and covariates. Figure (**a**) presents observed survival in the Experimental period (2008–2018), Figure (**b**) presents survival in the Experimental period (2008–2018), adjusted for lead time, and Figure (**c**) depicts the predicted survival in the Projection period (2019–2021), adjusted for lead time and covariates. To improve visibility, only the 95% confidence intervals for overall survival are shown, indicated by the shaded area.

First, we estimated the survival for women diagnosed with breast cancer invited to the programme and for those not invited as observed during the Experimental period (see Figure 4, left). These estimates were not adjusted for lead time or relevant confounders; therefore, the comparison between the two groups does not allow for causal interpretation. The invited group is divided into subgroups of screen-detected, interval, and non-attenders, where the screen-detected group has consistently the best survival, and non-attenders have consistently the worst survival.

In the next step, we adjusted the survival of screen-detected cases for lead time bias, as shown in the central graph of Figure 4. This adjustment resulted in a lower estimate of survival for screen-detected cases and, consequently, also for all cancer patients invited to the programme. Despite these adjustments, the two groups—those invited and those not invited—remain incomparable due to differences in the distribution of confounding variables.

Lastly, a Cox model was used to predict the survival for each subgroup in the Projection period. The estimated coefficients for the Cox model are presented in Appendix A. The predicted survival curves are adjusted for lead time bias and relevant confounding variables, matching the distribution observed during the Projection period. These predictions are unbiased under the model’s assumptions. As shown in Figure 4c, the survival rates for both cancer patients invited to the programme and those not invited have improved relative to the period from 2008 to 2018 (Figure 4a,b), reflecting advancements in cancer treatment and care. More importantly, the survival of cancer patients invited to the programme is higher compared to those not invited, indicating a general positive effect of cancer screening programmes on the whole population.

### 3.3. Years of Life Gained and Avoidable Deaths

**Figure 5:**
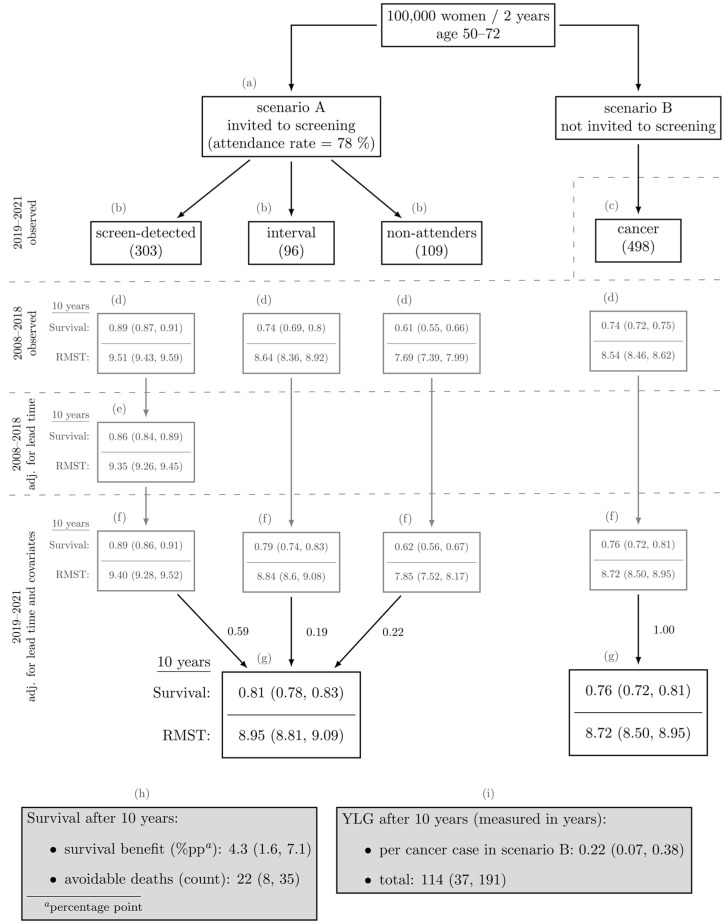
Flowchart comparing the survival probabilities and restricted mean survival times (RMSTs) of cancer patients between Scenario A and Scenario B (95% confidence intervals in the brackets). Each step is detailed in Section 2.2.4. (YLG—years of life gained.)

Both years of life gained and avoidable deaths depend on the number of individuals invited to the programme. To ensure the results are generalizable, we standardized them to a population size of 100,000 individuals over the 2-year period from 2019 to 2021 (the Projection period). We investigated how reality unfolds for this population under two different scenarios: in Scenario A, individuals at risk are invited to the programme exactly once, and in Scenario B, they are not. In Figure 5, we compare the two scenarios head-to-head. For transparency, we have included in the figure the observed survival (and RMST), as well as the lead time adjusted survival (and RMST). These metrics should not be directly compared between the two scenarios but are provided to illustrate how different sources of bias can affect the results. Only the results adjusted for both lead time and covariates provide a valid basis for comparing the two scenarios. Since we do not know which cancer patients in Scenario B would be screen-detected, we can only compare the survival of all cancer patients in Scenario A against the survival of all cancer patients in Scenario B. This comparison reveals that, after 10 years, cancer patients in Scenario A, being invited to the screening, have a 4.3 (95% CI 1.6, 7.1) percentage points higher survival probability (resulting in 22 (95% CI 8, 35) avoided deaths) and, on average, lived 0.22 (95% CI 0.07, 0.38) years longer per cancer case (which amounts to a total of 114 (95% CI 37, 191) years of life gained in population of 100,000 women) compared to those in Scenario B (women who are not invited). Note that the 95% confidence intervals do not include zero, meaning that the effect of the programme on 10-year survival is statistically significant at α = 0.05.

**Figure 6:**
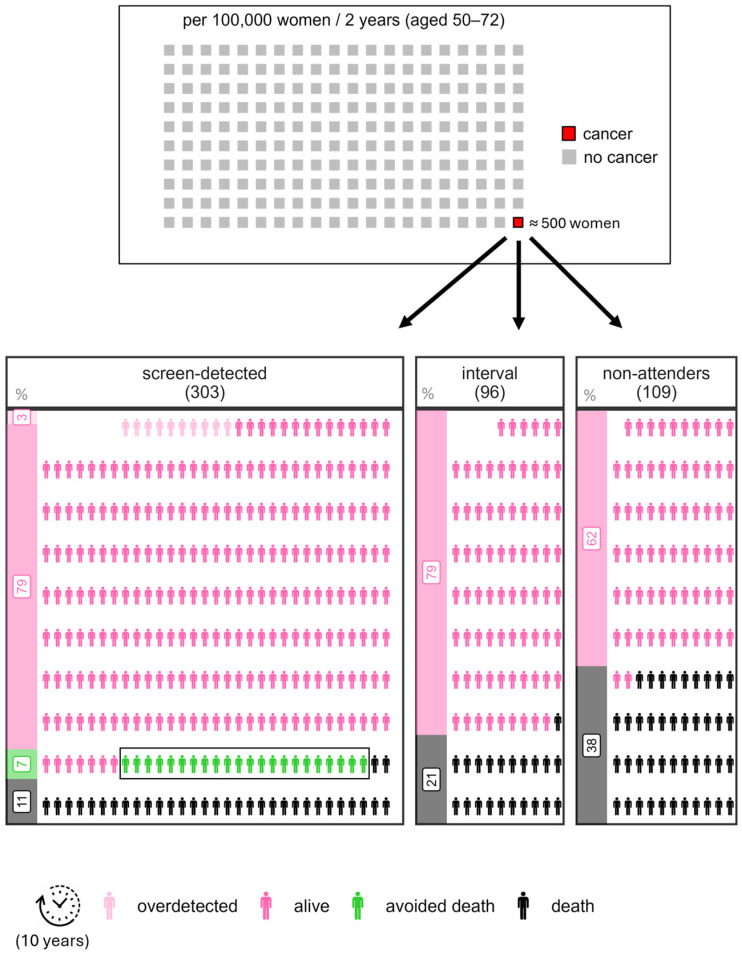
Infographic depicting results from the flowchart for Scenario A—Cancer cases in 100,000 women invited to screening. For this illustration all avoided deaths are attributed solely to the group of screen-detected cases. One box in the upper infographic corresponds to 500 women; one human silhouette in the lower infographic corresponds to one woman.

The results for Scenario A, as outlined in the flowchart (Figure 5), are visually depicted in an infographic (Figure 6). An additional simplification was made for the purpose of this illustration: specifically, all avoided deaths are assigned solely to the group of screen-detected cases. In our calculations, the avoidable deaths are calculated for Scenario A comparing to Scenario B, so in reality they cannot be attributed to a single subgroup of invited women.

## 4. Discussion

Evaluating outcomes of screening programmes represents quite some challenges [29,30,31]. One of the greatest challenges is using methodologically plausible and ethically acceptable methods which would enable comparisons of the outcomes between groups which would differ only by the intervention under study, that is, availability of breast cancer screening. In using survival as the measure of evaluating the benefits of cancer screening on the outcomes in the population, there are three main biases that strongly influence its use, namely lead time bias, length bias, and bias due to over-detection. Nevertheless, complex methodological approaches try to overcome the known biases by applying models to estimate different biases imposed on a person exposed to screening [12], and only some models are trying to correct for more than one bias at once. Recently, Vratanar and Pohar Perme proposed a new non-parametric estimator that allows for estimating the survival of cancer patients that would be screen-detected among those not included in the screening programme by applying estimations of lead time bias using the tumour growth model [21], corrected for confounding variables, and thus importantly reducing all of the above-mentioned biases [22].

Our study assesses the effectiveness of the Slovenian breast cancer screening program by comparing survival between invited and non-invited individuals in a modelled situation, estimating life years gained and avoidable deaths within 10 years as standardized measures for cross-country comparisons. Our results confirm that implementation of organized screening has a positive effect on the eligible population; in 498 cancer patients selected from 100,000 eligible women, the invited have a 4.3 (95% CI 1.6, 7.1) percentage points higher survival probability than comparable not-invited cancer patients, resulting in 22 (95% CI 8, 35) avoided deaths. On average, the breast cancer patients in the invited population each lived 0.22 (95% CI 0.07, 0.38) years longer within a limited time period of 10 years, which amounts to a total of 114 (95% CI 37, 191) years of life gained in the pool of 100,000 eligible women. We believe that years of life gained (YLG) is a more comprehensive and understandable measure for the general public and decision-makers, making it particularly valuable for public health communication [32]. It also evaluates impact over a life-time horizon and has a real-world meaning. To further enhance understanding, we have included an infographic that visually represents three distinct groups, clearly illustrating the lives saved in breast cancer cases due to the implementation of organized screening [31]. Furthermore, by using correction for lead time bias and the confounding variables, our results are comparable to similar calculations that could be performed in other settings.

As seen in the results section, we have calculated the survival for three groups of women—screen-detected, interval cancers, and non-attenders—which all add to the overall benefit of introduction of screening in a population. As expected, the worst survival is in the group of non-attenders (0.62; 95% CI 0.56, 0.67), which is significantly worse than if screening had not been introduced (0.76; 95% CI 0.72, 0.81). As previously known, non-attenders are a special group of people, with a lower health awareness and lower health system use [33,34,35]. This encourages providers of organized screening to give special attention to groups of people who do not respond to breast cancer screening invitations. Reasons for not attending cancer screening programmes could be very different in different countries and even within countries, where differences in income, education, socio-economic status, cultural norms, health status and health perception, understanding of the provided information, social support, and many others are most commonly studied and mentioned [27,36]. Our study does not focus on possible circumstances contributing to non-attendance, but by using the socio-economic index SI-EDI in a Cox model, we have tried to counterbalance reasons that could be connected to socio-economic status [37]. The greatest survival is observed in screen-detected cases (0.89; 95% CI 0.86, 0.91), which is significantly higher than in case of the non-invited, which equates to a scenario of no breast cancer screening programme (0.76; 95% CI 0.72, 0.81). This result further supports the fact that cancers detected and treated in earlier stages provide better outcomes. This is one of the key rationales behind the introduction of cancer screenings [2,3,36], and is also supported by findings in other countries. In our previous work [25], we calculated survival differences between invited and non-invited women during the roll-out period, aiming to minimize lead time bias by comparing different stages of disease, recognizing that earlier stages are more frequently detected through screening. However, comparing groups from different geographical areas, time periods of diagnosis, and varying age and socio-economic structures introduced selection bias, compounding the inherent biases of screening. In that study, the difference in 5-year net survival between attenders and non-attenders or non-invited women at localized and regional stages ranged from 4 to 10 percentage points [25].

One of the key strengths of our study is the availability of comprehensive nationwide real-world data, which serves as a robust foundation for calculating the parameters needed for modelling the benefits of organized cancer screening. The population-based data from our cancer and breast cancer screening registries are of high completeness and of excellent quality. This enables us to assess the impact of the phased implementation of screening across the entire population, as different segments were covered at varying times, providing valuable insights into its effectiveness. We have used the confounders that were readily available in the registry data. In further studies, additional confounding variables could be added (i.e., morphology), which could improve the modelling results.

In our study we have calculated parameters from the Experimental period to be used in a model for a control group in the Projection period from the real situation, which was possible due to the natural experiment of the 10-year roll out period of organized breast cancer screening in Slovenia. We have also considered the confounding variables that differed in the invited and not invited population in the Experimental period on real-world data. By using two total populations for comparison, we avoided the length time bias.

By applying the biological tumour growth model, we have calculated the lead time introduced into breast cancer patient data by screening. This enabled us to observe and compare groups of people from a common starting point to avoid lead time bias. For calculating the lead time bias, we used an approach based on Abrahamsson’s model [21] applied to Slovenian cancer-screening data. We applied model parameters derived from the Swedish dataset of a breast cancer screening programme as we could not estimate the parameters from the Slovenian data due to the lack of tumour size measurements for interval cases. This is a key limitation of our study, as differences in tumour growth dynamics between populations could introduce bias. Obtaining the missing data would allow us to estimate these parameters directly, leading to more reliable lead time estimates. The calculated lead time was 1.23 years, which is slightly lower than the calculation on the Swedish screening data (the mean there was 1.9 years). This could be due to the fact that our Experimental period consisted of more women who were screened for the first time, no matter their age, since the screening programme had just been spreading out to the whole country. In the first round of screening, it is foreseen that more cancers are discovered and they are of greater size [27], compared to well-established screening programmes, such as the Swedish one, which has been operating nation-wide since 1986 [4], and thus invites mostly women that have already been screened in previous rounds. We expect that our lead time will eventually lengthen.

We have shown results for all steps of the process—adjustment for lead time bias alone and for lead time bias and confounding factors, where these metrics should not be directly compared between the two applied scenarios but are provided to illustrate how different sources of bias can affect the results. This adds to the pool of evidence how survival calculations could be improved and become more useful in the evaluation of screening programme implementation.

## 5. Conclusions

Comparison of screening outcomes is difficult in many aspects. Using survival, which needs less time to observe than mortality, the main concern is the selection of a control group and addressing the known biases, such as lead time bias, length bias, and over-detection bias. In this study we have combined several well-established approaches that are usually used in different areas of epidemiological research, but not in monitoring cancer screening benefits. That means that we have applied a newer, more complex approach to calculate survival probabilities using real-world data for Slovenian women diagnosed with breast cancer. We have considered the biases, where the lead time bias was calculated individually taking into account the individual size of the tumour at the time of diagnosis, and also correcting for covariates between the comparison groups, to calculate the benefit of the introduction of an organized screening programme compared to a population in a hypothetical situation where no screening would have been available for the same population. We believe that such comparison of outcomes has a sounder ground, provides possibilities of evaluation of survival benefits of fully implemented screening programmes, and could be used in further studies of screening outcomes, also in the context of the introduction of more personalized approaches to screening. The calculation of years of life gained in the context of screening programme implementation provides additional opportunities for communicating the benefits of breast cancer screening to the non-expert community, since this measure is well established in other epidemiological studies, but also more understandable to policy-makers and other lay public.

## Data Availability

Detailed results are available from the corresponding authors upon reasonable request.

## Abbreviations

The following abbreviations are used in this manuscript:

SI-EDI	Slovenian version of the European Deprivation Index
RMST	restricted mean survival time
YLG	Years of life gained

## Appendix A

In our work, we fitted the Cox proportional hazards model to the data from the Experimental period. The following covariates were included in the model: age at diagnosis (adjusted for lead time), date of diagnosis (adjusted for lead time), SI-EDI (entered into the model as a set of indicator variables with 1 being a reference category), and detection mode (entered into the model as a set of indicator variables with “non-invited cancer” being a reference category). The relationship between hazard and age, as well as the date of cancer diagnosis, was modelled using restricted cubic splines. The estimated coefficients in the Cox proportional hazards model are shown in Table A1.

**Table A1:** Summary of Cox proportional hazards model.

Variable	Coefficient	Std. Error	*p*-Value
Age at diagnosis	1.059 × 10^−2^	1.038 × 10^−2^	0.308
Age at diagnosis	3.227 × 10^−2^	1.164 × 10^−2^	0.006
Date of diagnosis	−9.368 × 10^−3^	1.678 × 10^−2^	0.577
Date of diagnosis	−1.509 × 10^−2^	2.379 × 10^−2^	0.526
SI-EDI	6.069 × 10^−2^	1.830 × 10^−2^	9.100 × 10^−4^
Screen-detected	−0.670	0.094	8.179 × 10^−13^
Interval	−0.044	0.117	0.710
Non-attenders	0.527	0.085	5.174 × 10^−10^
